# SENSITIVITY OF SELF-REPORTED COMORBIDITIES COMPARED TO MEDICARE CLAIMS IN OLDER ADULTS WITH TRAUMATIC BRAIN INJURY

**DOI:** 10.1093/geroni/igz038.1796

**Published:** 2019-11-08

**Authors:** Aparna Vadlamani, Jennifer Albrecht

**Affiliations:** University of Maryland Baltimore, Baltimore, Maryland, United States

## Abstract

Patient reported history of comorbid illness may be the only information available to the treatment team during an acute injury admission. Nevertheless, acute injury, particularly traumatic brain injury (TBI) which affects cognition, may decrease the patient’s ability to accurately report medical history. Thus, the objective of this study was to evaluate the accuracy of patient-reported comorbid illness burden compared to the patient’s Medicare administrative claims. Records of older adults treated for TBI at an urban level 1 trauma center 2006-2010 were linked to their Medicare administrative. Comorbidities were recorded in Medicare claims based on ICD9 codes and were reported in the trauma registry (TR) based on patient medical history recorded by a physician or nurse. Prevalence of each of the following comorbidities was calculated using information from the TR and claims: Alzheimer’s disease and related dementias, chronic kidney disease, COPD, heart failure, diabetes, depression, stroke, and hypertension. Sensitivity of each patient-reported comorbidity was calculated using Medicare claims as the gold standard. We identified patient factors associated with accurate self-report using logistic regression. Among 408 older adults with TBI that linked to their Medicare claims, prevalence of each comorbidity was higher in Medicare claims compared to the TR, except for hypertension. Sensitivity for detecting these comorbidities using the TR ranged from 2% to 68%, with the highest sensitivity observed for hypertension. Older age and race were predictors of less accurate reported medical history. Reconciling self-reported patient history of these comorbidities with those reported in claims can better inform decisions regarding treatment.

